# Optimal filtering strategies for task-specific functional PET imaging

**DOI:** 10.1177/0271678X251325668

**Published:** 2025-04-02

**Authors:** Murray Bruce Reed, Magdalena Ponce de León, Sebastian Klug, Christian Milz, Leo Robert Silberbauer, Pia Falb, Godber Mathis Godbersen, Sharna Jamadar, Zhaolin Chen, Lukas Nics, Marcus Hacker, Rupert Lanzenberger, Andreas Hahn

**Affiliations:** 1 Department of Psychiatry and Psychotherapy, 27271 Medical University of Vienna, Vienna, Austria; 2 Comprehensive Center for Clinical Neurosciences and Mental Health (C3NMH), 27271 Medical University of Vienna, Vienna, Austria; 3 Monash Biomedical Imaging, 2541 Monash University, Melbourne, Victoria, Australia; 4 School of Psychological Sciences, Monash University, Melbourne, Victoria, Australia; 5 Department of Data Science and AI, 2541 Monash University, Melbourne, Victoria, Australia; 6 Department of Biomedical Imaging and Image-guided Therapy, Division of Nuclear Medicine, Medical University of Vienna, Vienna, Austria

**Keywords:** Functional Positron Emission Tomography (fPET), [^18^F]2-fluoro-2-deoxy-D-glucose ([^18^F]FDG), non-local means (NLM), highly constrained back projection (HYPR), filtering, MRI-Markov Random Field (MRI-MRF)

## Abstract

Functional Positron Emission Tomography (fPET) is an effective tool for studying dynamic processes in glucose metabolism and neurotransmitter action, providing insights into brain function and disease progression. However, optimizing signal processing to extract stimulation-specific information remains challenging. This study systematically evaluates state-of-the-art filtering techniques for fPET imaging. Forty healthy participants performed a cognitive task (Tetris®) during [^18^F]FDG PET/MR scans. Seven filtering techniques and multiple hyperparameters were tested: including 3D and 4D Gaussian smoothing, highly constrained backprojection (HYPR), iterative HYPR (IHYPR4D), MRI-Markov Random Field (MRI-MRF) filters, and dynamic/extended dynamic Non-Local Means (dNLM/edNLM). Filters were assessed based on test-retest reliability, task signal identifiability (temporal signal-to-noise ratio, tSNR), spatial task-based activation, and sample size calculations were assessed. Compared to 3D Gaussian smoothing, edNLM, dNLM, MRI-MRF L = 10, and IHYPR4D filters improved tSNR, while edNLM and HYPR enhanced test-retest reliability. Spatial task-based activation was enhanced by NLM filters and MRI-MRF approaches. The edNLM filter reduced the required sample size by 15.4%. Simulations supported these findings. This study highlights the strengths and limitations of fPET filtering techniques, emphasizing how hyperparamter adjustments affect outcome parameters. The edNLM filter shows promise with improved performance across all metrics, but filter selection should consider specific study objectives and resource constraints.

## Introduction

Functional Positron Emission Tomography (fPET) has become a powerful tool, enabling researchers and clinicians to investigate the intricate details of biological processes at the molecular level *in vivo.*^1,2^ This capability to visualize and quantify task-driven dynamic changes in molecular activity has far-reaching implications for understanding brain function, disease progression, and monitoring treatment efficacy. Although research into fPET began almost a decade ago,^3,4^ rapid methodological advances in the field have led to improvements in temporal resolution. Commencing with more conventional PET framing times of 60 s, fPET quickly progressed to 30 s^1,2,5^ and more recently 16 s.^6^ Notably, a recent study unveiled acute temporal changes in the fPET signal after task performance using 3 s frames, which was previously indiscernible at lower temporal resolutions.^7^ This breakthrough was achieved, in part, through specialized filtering techniques designed to enhance the temporal signal to noise ratio (tSNR), while preserving the acute task-specific changes. Given the inherently low tSNR of PET and the recent interest and rapid advances in high temporal resolution imaging, the demand for enhancing both spatial and temporal resolution in fPET data is becoming increasingly imperative. However, the potential of high-resolution fPET is fundamentally limited by challenges related to spatial^8^ and temporal resolution.^9^ The precise localization of molecular events and the ability to capture rapid changes over time are essential for extracting clinically accurate and meaningful information.

Despite the considerable progress in the methodology behind fPET, achieving optimal spatial and/or tSNR remains an ongoing challenge. The need for improved resolution is emphasized by the complexities of biological systems, where rapid and subtle changes are often the key indicators of physiological and pathological processes. As such, the investigation and optimization of filtering techniques used to enhance spatial and temporal resolution in fPET is crucial for unlocking its full potential.

Existing methods to enhance fPET data predominantly involve 3D filtering techniques. The most common and widely used approach is Gaussian smoothing, employed not only in fPET,^10,11^ but also in structural and functional magnetic resonance imaging (fMRI).^12,13^ Other 3D filtering techniques exist, such as highly constrained backprojection (HYPR),^14^ dynamic non-local means^15^ (dNLM) and a more recent technique incorporating anatomical knowledge to enhance the image using a Bowsher-like prior.^16^ Each of these techniques has its own advantages and limitations. Although NLM filtering including the temporal domain has been explored in fMRI^17^ and in PET for denoising low-count frames^18^ its rigorous evaluation in the context of fPET is missing. Other techniques like Gaussian smoothing and HYPR have seen recent improvements by incorporating a temporal component.^19–21^

While each filtering technique has been shown to improve spatial and/or tSNR when compared to a standard approach using either simulations or phantom data, a comprehensive assessment of the most well-known techniques on the same real-world dataset using multiple test metrics has yet to be performed. The primary objective of this study was to assess the efficacy of various filtering techniques in optimizing fPET task imaging results, with a specific focus on practically relevant parameters such as test-retest reliability, tSNR, group level spatial effects and sample size estimation. By systematically evaluating and comparing different filtering techniques in a simulated and practical manner, we aim to identify the most promising approaches for enhancing the quality of fPET images.

## Materials and methods

For this retrospective analysis, data from our previous test-retest study was used.^22^ Thus, full details about the study design and data acquisition can also be found there and in related work.^1,11^ To minimize potential diurnal variations in the test-retest data, participants' scans were scheduled at the same time of day whenever possible. The mean relative time difference between the two measurements was 01:09:48 ± 01:49:23 (hh:mm:ss).

## Participants

Data from 40 healthy participants (20 male, mean age ± SD: 23.0 ± 3.4 years, all right-handed) were used, and for 20 participants, data for test-retest reliability analysis was available (10 male, 23.1 ± 3.1 years). All participants underwent a routine medical investigation at the screening visit including electrocardiography, blood tests, neurological and physiological examination, and a urine drug test. Psychiatric disorders were ruled out with the Structural Clinical Interview DSM-IV conducted by an experienced psychiatrist. Female participants additionally underwent a pregnancy test at the screening visit and before each PET/MRI measurement. Participants had to fast for at least four hours prior to the scan, including the consumption of sweetened beverages and caffeine.^23^ Exclusion criteria included current or previous neurological, somatic or psychiatric diseases, current breastfeeding or pregnancy, left-handedness, substance abuse, MRI contraindications, past participation in a study with ionizing radiation exposure and regular experience playing puzzle games i.e. Tetris® or similar. Due to radiation protection, participants above 100 kg were also excluded. After a detailed explanation of the study protocol, all participants gave written informed consent. Participants were insured and reimbursed for their participation. The study was approved by the Ethics Committee of the Medical University of Vienna (ethics number: 1479/2015) and all procedures were carried out in accordance with the Declaration of Helsinki. The study was registered at ClinicalTrials.gov (ID: NCT03485066).

## Cognitive task

Participants were required to play an adapted version of Tetris®, including 2 levels of difficulty to induce various levels of cognitive load. Participants were required to perform all actions using only their right hand. To familiarize themselves with the task and the controls, participants completed a 30 s training of each condition before each scanning session. A more detailed description of the task can be found in.^22,24^

## Study design

The experimental protocol aimed to assess cognitive tasks using a combination of continuous task performance and a conventional block design. The session started with the acquisition of a structural T1-weighted image. Thereafter, [^18^F]FDG was administered in a bolus + constant infusion protocol. The initial baseline was 8 minutes of rest. Participants then performed four task conditions of varying difficulty (6 min each, 2 easy, 2 hard in a pseudo-randomized order), followed by a 5-minute rest condition where subjects were instructed to look at a crosshair and let their thoughts wander. Additional data acquired for different purposes were not used in this study (ASL, BOLD and DTI sequences). The total scan time was 100 minutes, representing a typical duration for PET studies. See Hahn et al. for a more detailed description.^1^ Data for test-retest analysis were acquired 4.2 ± 0.7 weeks after the first scan.

## Data acquisition and blood sampling

The radiotracer [^18^F]FDG was synthesized each measurement day at the Department of Biomedical Imaging and Image guided Therapy, Division of Nuclear Medicine, Medical University of Vienna. Simultaneous to fPET start, [^18^F]FDG was administered via a cubital vein as a 1 minute bolus followed by constant infusion for 51 minutes with an infusion pump (Syramed mSP6000, Arcomed, Switzerland, dosage: 5.1 MBq/kg, bolus speed: 816 ml/h, infusion speed: 42.8 ml/h, bolus-infusion ratio of activity: 20:80%), which was placed in an MR-shield. fPET data was acquired in list-mode using a Siemens 3T mMR scanner (Erlangen, Germany), enabling the retrospective definition of frame lengths during reconstruction. The mMR has a spatial resolution of 4.3 mm full width at half maximum (FWHM) when measured 1 cm from the center of the field of view.^25^

A T1-weighted structural image was acquired with a magnetization prepared rapid gradient echo (MPRAGE) sequence prior to radiotracer administration (TE/TR = 4.21/2200 ms, voxel size = 1 × 1 ×1.1 mm, matrix size = 240 × 256, slices = 160, flip angle = 9°, TI = 900 ms, 7.72 min). The image was used to rule out severe structural abnormalities, for attenuation correction^26^ and spatial normalization to MNI space.

Prior to each PET/MRI measurement, the individual fasting blood glucose level was measured as an average of a triplicate. Arterial blood samples were drawn from a radial artery throughout the radiotracer administration (time points: 3, 4, 5, 14, 25, 36 and 47 min after infusion start) and were timed not to interfere with task performance and the MRI acquisition. Blood samples were processed as previously described.^11^ In short, whole blood activity and plasma activity after centrifugation were measured in a gamma-counter (Wizard2, 3″; Perkin Elmer, USA). The whole blood curve was linearly interpolated and resampled to match the time points of the reconstructed fPET frames. The plasma to whole-blood ratio was averaged across time points. The whole blood curve was then multiplied with the mean plasma-to-whole-blood ratio to obtain an arterial input function for absolute quantification.

## Preprocessing and quantification

All fPET data was reconstructed using an Ordinary Poisson – Ordered Subset Expectation Maximization Algorithm (OP-OSEM), set at 3 iterations and 21 subsets. The output image contained a matrix size of 344 × 344 with 127 slices and a voxel size of 2.09 ×2.09 × 2.03 mm. The reconstructed data was binned into 104 frames of 30 s each. Standard corrections, including dead time, decay, and scatter, were applied, and attenuation correction was performed using a pseudo-CT approach based on the structural MRI acquired during the initial measurement.^26^

Preprocessing and quantification followed established procedures from previous studies.^1,4,5^ Specifically, SPM12 (https://www.ﬁl.ion.ucl.ac.uk/spm) was utilized for head movement correction (quality = best, registered to mean image), spatial normalization to MNI space using the structural MRI. The mean PET image was coregistered to the structural MRI, and the resulting transformations were applied to the dynamic fPET data. Once normalized, the data were processed using different techniques, see filter assessment below. The processed data was subsequently masked using a grey matter mask (SPM12 tissue prior, thresholded at 0.1), and a low pass filter with a cutoff frequency of half the task duration was applied to the time course of each voxel.

A general linear model (GLM) was employed to distinguish between task-specific and baseline metabolism, using four regressors: baseline, easy task block, hard task block and the first principal component of the six movement regressors obtained during movement correction.^4^

The Gjedde-Patlak plot was employed to derive the influx constant (K_i_), with linearity assumed after 15 min after tracer application. This resulted in three separate K_i_ maps for rest, easy, and hard. Finally, the cerebral metabolic rate of glucose (CMRGlu) was quantified using the lumped constant of 0.89.^27^ For a more detailed description please see.^22^

## Filter assessment

In this work we aimed to assess the most recognized PET filtering techniques which were implemented in MATLAB unless otherwise specified. These filters include:

### 3D Gaussian filter

Is the most commonly used spatial smoothing technique in image processing. It operates by convolving the image with a three-dimensional Gaussian kernel. The convolution process assigns a weighted average to each voxel in the image, with the weights determined by the Gaussian distribution. This smoothing helps reduce noise and emphasize larger-scale features in the data.^28^ Here we used the SPM function with a smoothing kernel with a FWHM of 6, 8 and 10 mm and a mean averaging volume of 7.5, 17.5 and 34.5 voxels.

### 4D Gaussian filter

Which extends on the concept of the 3D Gaussian filter to four dimensions, incorporating time as an additional dimension. This filter is particularly relevant in the context of dynamic imaging data, such as functional imaging over time. A spatial kernel of 8 mm FWHM and a Gaussian temporal window of 3, 5 and 7 frames (e.g. 2 before and 2 after selected frame; 2.5 min) were used. This yielded a mean averaging volume of 22.2, 36.75, 52.15 voxels.

### HYPR filter

Is an advanced filtering technique designed to improve spatial resolution in medical imaging. It operates by incorporating constraints into the back-projection process during image reconstruction or preprocessing. These constraints, derived from both the acquired data and prior knowledge, guide the reconstruction algorithm to produce images with enhanced spatial details. HYPR can also be utilized in post-processing, where instead of back-projection, a composite (i.e., temporally summed) image with higher SNR from later time points is used to extract spatial features, which are then combined with individually smoothed frames to preserve spatial features present in the composite while (ideally) minimally affecting temporal dynamics. The spatial features are extracted from the composite by dividing the unsmoothed composite by the smoothed composite. HYPR is particularly useful in scenarios where high spatial resolution is crucial, such as in the case of small anatomical structures.^14^ In this work, we used the post-processing version of the HYPR filter which was applied with a 6, 8 and 10 mm FWHM kernel and a mean averaging volume equal to that of the 3D Gaussian filter.

### Iterative 4D HYPR (IHYPR4D) filter

Represents an extension of the HYPR filter combined with a 4D Gaussian filter, which incorporates both spatial and temporal constraints, while the iterative step reduces errors during the filtering process through multiple iterations.^21^ Parameters were selected from.^19^ In summary, the segmentation of homogenous regions from the mean fPET image was performed using k-means clustering where k was set to 30 for feature extraction prior to running IHYPR4D. The number of iterations were set to 4 and the smoothing kernel was set using the same parameters as the 4D Gaussian smoothing also yielding the same mean averaging volume.

### MRI-Markov random field (MRI-MRF)

The MRI-MRF prior, a modified Bowsher-like technique, enhances fPET image quality by incorporating anatomical information from coregistered MRI. This technique utilizes a continuous weighting scheme, patch-based similarity, and smoothly-decaying function which contribute to improved identification of brain activations.^16^ Here we tested three different MRF neighborhood width parameters L = 6 mm, 10 mm and 14 mm, as used in.^16^ Where the neighborhood refers to a spatial arrangement of nearby voxels around a central voxel. The mean averaging volume for each L-parameter is 7.5, 34.5 and 76.7 voxels.

### dNLM filter

Is an extension of the traditional non-local means filter, adapted for time-series imaging data. The non-local means approach involves averaging pixel values based on similarity patterns in the image. In the dNLM, this concept is extended to capture temporal correlations over the entire time course in addition to spatial similarities. By considering both spatial and temporal information, the dNLM filter aims to preserve fine details in the data while effectively reducing noise in dynamic imaging sequences.^15^ The following parameters were used: a search window of D = 11 voxels and a patch size of 3 × 3 × 3 voxels.^18,29^ A post smoothing 5 mm FWHM was used for better comparison to the other techniques, as this yields a total filter kernel of approximately 8 mm FWHM.

### edNLM filter

Is a further extension of the dNLM by capturing only correlations over a short duration, similar to a sliding window approach. Thus, this method dynamically adjusts the spatial selection of voxels in the time domain. This means that the voxel neighborhood used for denoising varies over time, leading to time-changing voxel admixtures. While this aims to preserve acute (e.g., task-induced) signal changes while reducing noise,^7^ it also results in a temporally adaptive smoothing process that differs from conventional 3D NLM approaches. To incorporate a temporal parameter we used patch sizes of −3^3^ and 5^3^ voxels and also added a 4^th^ dimension to the patch size of 3, 5 and 7 frames or 1.5, 2.5 or 3.5 min. Here a 5 mm FWHM post smoothing was also used. As NLM averaging volumes are inhomogeneous in these cases, the average volume was not computed for comparison.

## Statistical analysis

A rigorous analysis of the acquired data was conducted employing a multifaceted approach to ensure comprehensive insights of each filter’s properties of both spatial and temporal dimensions. For all tests three regions of interest (ROIs) were selected from previous analyses on the same dataset, which represent a robust task-activation assessed via a conjunction of three imaging modalities.^1^ These regions comprise the frontal eye field (FEF), intraparietal sulcus (IPS) and the occipital cortex (OCC).

The Intraclass Correlation Coefficient (ICC) was utilized to assess the reliability and consistency of the observed effects between two repeated fPET measurements. Additionally, the individual tSNR was computed to gauge the robustness of the signal over time. This provides information about the ability to identify stimulation-induced changes in the presence of noise and, thus, an insight into the temporal information captured by the filtering techniques. At the group level, peak and mean task-specific t-values were compared to quantify the strength of neural responses and model fit after each filter technique. T-tests were corrected for multiple testing using Gaussian random field theory as implemented in SPM12 and the threshold for significance was set at p < 0.05 family-wise error (FWE)-corrected at the cluster-level following p < 0.001 uncorrected at the voxel-level and separately at the peak FWE-corrected p < 0.05. A power analysis was conducted to determine the requisite sample size for detecting a significant effect in a prospectively planned study (one sample case, α = 0.05, Power =0.95, two-tailed). The effect size for each filtering approach was assessed by utilizing the mean and standard deviation of task-induced CMRGlu clusters, while accounting for multiple comparisons, i.e., number of voxels, via the Bonferroni adjustment method (p < 2.585 * 10^−7^). CMRGlu clusters were determined using each filter’s quantified fPET maps where an overlap with the aforementioned three specified ROIs occurred. The 3D Gaussian filter was used as a reference for filter comparisons, since it represents the most commonly employed approach in functional neuroimaging.

## Simulation

To evaluate the performance of each filter type with respect to a known ground-truth, fPET task activations were simulated as follows. The irreversible two-tissue compartment model with rate constants from previous work^7^ was applied, and a mean arterial input function derived from 51 subjects was used^24^ to compute a representative time activity curve. To characterize baseline time series within the entire brain, this TAC was scaled by a mean baseline beta map. The task regressor was modeled as a ramp function with a slope of 1 kBq/frame during two distinct task blocks similar to the original data, and 0 kBq/frame at all other times. Task effects within the entire brain were then generated by scaling this regressor with the average task-specific beta map.^7^ The resulting task-specific patterns were subsequently added to the baseline activity. Finally, five different levels of Gaussian noise levels (10, 20, 50, 75 100%) were incorporated into the data to simulate imaging conditions. Repeating the last step 5 times resulted in 25 simulated fPET images (i.e., 5 data sets for each of the 5 noise levels). A GLM was employed to distinguish between simulated task and baseline activity, using the same regressors as mentioned above in section Preprocessing and quantification. The GLM task-beta maps were then assessed.

## Results

**Figure 1.**
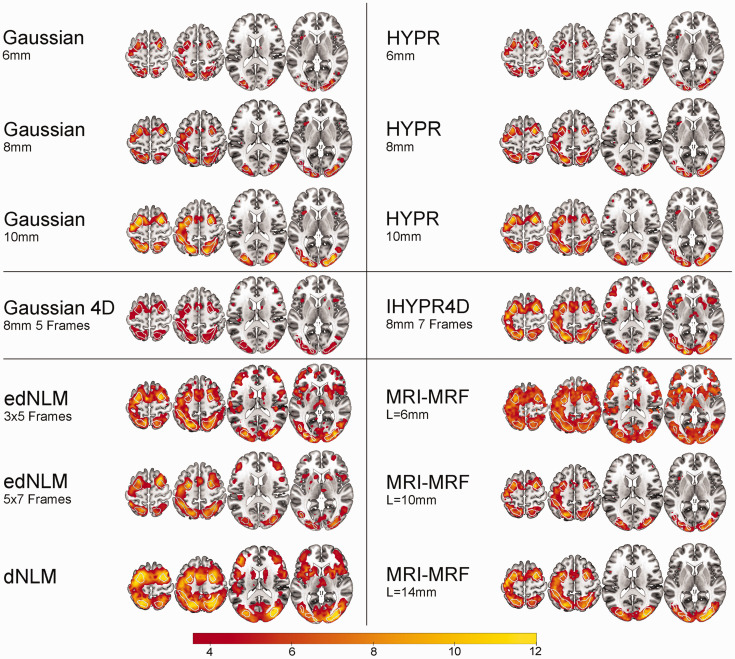
Spatial overview of task-performance (Hard > baseline) for the best performing hyperparameters per filter technique. Group level statistical analysis was carried out for the hard task condition (n = 40), corrected at p < 0.05 FWE cluster level, after p < 0.001 uncorrected voxel level. White outlines indicate the 3 task-active regions. The color bar represents the mean T-values.

**Figure 2.**
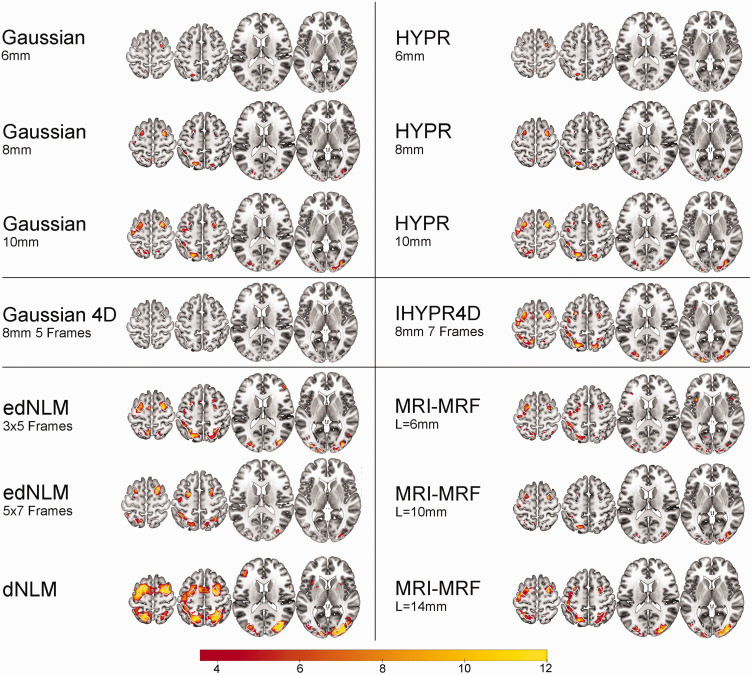
Spatial overview of task-performance (Hard > baseline) for the best performing hyperparameters per filter technique. Group level statistical analysis was carried out for the hard task condition (n = 40), corrected at p < 0.05 FWE voxel level. White outlines indicate the 3 task-active regions. The color bar represents the mean T-values.

An overview of the best performing intra-method filter’s performance for each test can be found in Table 1. A more detailed overview of all filter’s regional performance can be found in table S1–4. Group activation maps at cluster level correction (Figure 1) and voxel level correction (Figure 2) for the best performing hyperparamters per filter technique were created. While both Gaussian filtering techniques exhibit very low runtime on our data (mean runtime: ∼30 s), the MRI-MRF (mean runtime: 14–16.5 h) and NLM (mean runtime: 7.9 h) filters require substantially longer processing times. The NLM filters were processed on a single core for fair comparison, although parallelization is available, halving the runtime per core. The HYPR and IHYPR4D filter displayed moderate mean runtimes of 4 and 5 min per dataset, respectively.

## Test-retest reliability

The comparison of ICC values revealed that the edNLM and HYPR filter showed notable improvements to task-based test-retest reliability, compared to other filters. Among the NLM-based filters, edNLM 3 × 5 exhibited on average the highest ICC (0.61), marking a relevant enhancement over the 3D Gaussian smoothing. In contrast, the 4D Gaussian filters and in particular the MRI-MRF L = 6 yielded the lowest ICC values, with the 4D Gaussian (7 frames) dropping as low as 0.26 and MRI-MRF L = 6 to 0.09, representing a substantial decrease in reliability. The IHYPR4D (3 and 5 frames) displayed moderate performance but was outperformed by its 7 frame variant, which achieved an ICC of 0.56, comparable to the edNLM family (Table 1, S1). Meanwhile, dNLM achieved an ICC of 0.50, lower than the reference 3D Gaussian but still within a competitive range.

## Temporal signal-to-noise ratio

Most filter approaches, excluding the 4D Gaussian smoothing and the MRI-MRF, displayed similar or improved tSNR values relative to the 3D Gaussian filter (Table 1, S2). The IHYPR4D 7 frames and edNLM 5 × 7 achieved the highest tSNR (14.27, 13.74), exceeding all other methods, including the 3D Gaussian 6 mm (12.28). Other edNLM configurations (3 × 3, 3 × 5, 3 × 7 and 5 × 3) also showed consistent gains in tSNR, underscoring the strength of NLM-based approaches in preserving temporal signal fidelity. HYPR filters showed competitive performance, with tSNR values ranging from 11.91 to 12.36, closely aligning with the 3D Gaussian. MRI-MRF exhibited mixed results: while the 10 mm configuration performed well (tSNR = 12.00), the 6 mm and 14 mm configurations lagged behind (8.50 and 10.61, respectively). The 4D Gaussian, regardless of the temporal points (3, 5, or 7 frames), consistently underperformed, with tSNR values ranging between 10.25 and 11.61.

## Spatial task-based activation

Spatial activation metrics highlighted substantial differences across filter techniques. The edNLM 3 × 5 filter produced a high mean (6.22) and peak t-values (14.18), signifying superior performance in enhancing task-based activation. The edNLM 5 × 7 also performed exceptionally well, achieving mean and peak t-values of 6.66 and 14.23, respectively. Among the Gaussian filters, larger kernel sizes (8 mm and 10 mm) showed incremental improvements over the 6 mm variant, with the 10 mm filter achieving a peak t-value of 11.54. MRI-MRF displayed high variability: while the L = 14 mm configuration achieved strong peak t-values (13.12), whereas the 6 mm version underperformed. HYPR filters performed similar to 3D Gaussians, with peak t-values ranging from 8.38 to 11.93, but they did not reach the levels observed with the edNLM filters. The 4D Gaussian filter consistently produced the lowest spatial activation values, reinforcing its suboptimal performance in this domain (Table 1, S3-4). Task-based spatial activation maps for each filtering technique can be found in Figures 1 and 2.

## Temporal differences

**Figure 3.**
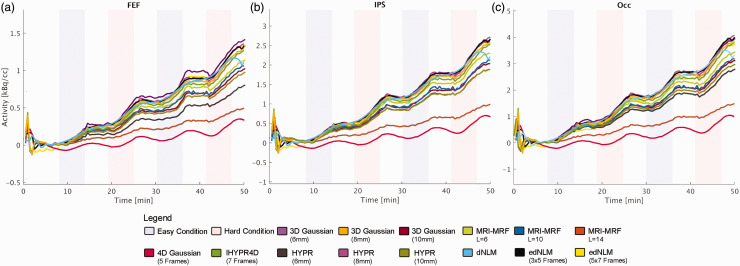
Illustration of the average task regressors’ time course for the best performing hyperparamters per filtering technique for a subset of participants whose task difficulty was ordered as easy-hard-easy-hard (n = 10), extracted from each task-positive region of interest. Excluding the MRI-MRF L = 14 (dark orange) filter, each filter successfully captured the task-induced increases in glucose metabolism. The MRI-MRF L = 10 (dark blue) and 4D Gaussian (red) filters exhibited a notable detrimental impact on baseline uptake (supplemental figure 1) and thus also task effects, evident in the flatter lines, pronounced to a much lesser degree in the MRI-MRF L = 10 (dark blue). Filters such as HYPR (pink), IHYPR4D (green), and edNLM (black and light yellow) displayed uptake profiles most akin to the 3D Gaussian filter (dark yellow), with edNLM exhibiting the least noise. Similarly, dNLM (cyan) exhibited a high signal-to-noise ratio akin to edNLM, albeit with attenuated task-specific effects. These regions included the (a) frontal eye field (FEF), (b) intraparietal sulcus (IPS) and (c) the occipital cortex (OCC).

The temporal dynamics of each filtering technique were scrutinized through the analysis of the task-specific fPET signal, as illustrated in Figure 3, supplemental figures 1 and 2. Notably, the MRI-MRF L = 14 and Gaussian 4D filters exhibited a tendency to overly smooth the signal, which is in line with the lower tSNR for these two filters (Table 1). The MRI-MRF L = 14, while capturing more acute changes in the PET signal, tended to excessively smooth out the overall task trend (Figure 3). Conversely, the Gaussian 4D filter demonstrated an opposite effect, preserving the general task effect but smoothing the acute changes excessively (Figure 3). In contrast, the HYPR, and MRI-MRF L = 10 filters demonstrated a better temporal alignment with the Gaussian 3D signal, showing reduced peaks while maintaining signal stability, see Figure 3. The dNLM filter exhibited a considerably smoother time course (i.e., small difference in the TAC slope between rest and task periods) compared to the edNLM, HYPR, and MRI-MRF L = 10 filters. The edNLM (3 × 5 frames) filter, presented a time course with reduced noise and better preserving the amplitude of task-induced changes. Interestingly, the MRI-MRF L = 14 was observed to underestimate tracer uptake also for the baseline condition when compared to other filtering techniques, as depicted in supplemental figure 1.

## Sample size calculation

The power analysis of the best performing filter variants revealed varying sample size requirements across filter techniques. The 3D Gaussian filter (8 mm) served as the reference, requiring 13 participants, while the 10 mm configuration reduced the requirement to 12 [92.3%]. The edNLM (3 × 5 Frames) filter was the most efficient, needing only 11 participants [84.6%]. Conversely, MRI-MRF L = 6 mm required the largest sample size, with 33 participants [253.8%], highlighting its limited performance. Intermediate filters, such as MRI-MRF L = 10 mm, HYPR 6–8 mm, and IHYPR4D (7 Frames), required 15–16 participants [115.4–123.1%], offering a balance between efficiency and performance. The 4D Gaussian (5 Frames) filter exhibited the highest inefficiency among Gaussian-based methods, requiring 19 participants [146.1%], see Table 1 for an overview.

## Simulations

**Figure 4.**
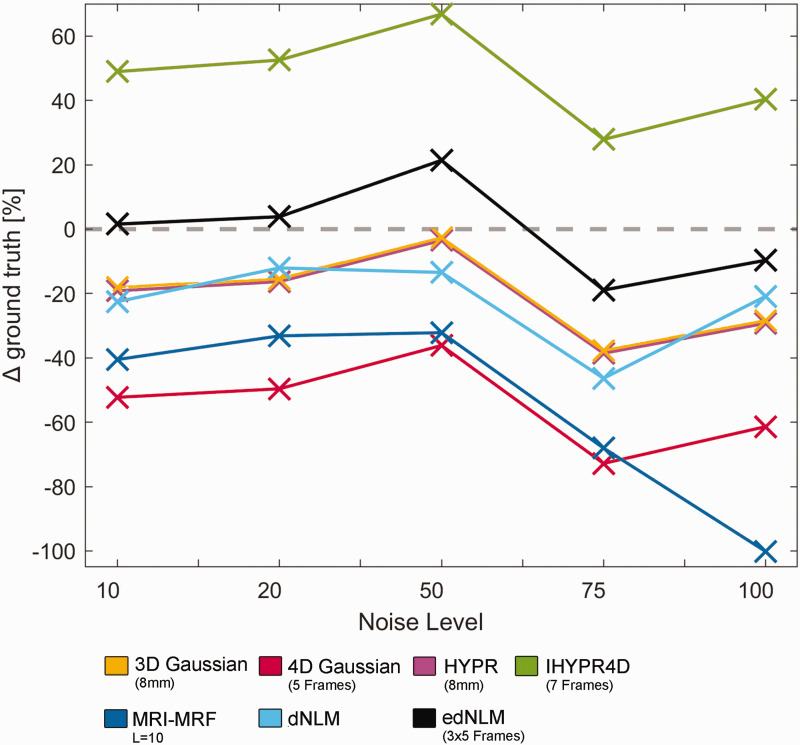
Overview of the average performance of various filtering techniques across different noise levels ranging from a multiplicative factor of 10 to 100, based on five simulation iterations for each noise level. This was measured by the deviation in the GLM output from the ground truth, averaged across regions. The HYPR (pink) and 3D Gaussian (yellow) filters demonstrated similar performance across all noise levels, while the MRI-MRF (dark blue) and dNLM (cyan) filters performed slightly worse. All these methods underestimated the simulated activation compared to the ground truth. The 4D Gaussian filter (red) showed the largest underestimation, whereas the IHYPR4D (green) filter tended to overestimate the ground truth. Among the evaluated methods, the edNLM (black) filter performed the best, closely approximating the ground truth across all noise levels. Ground truth is depicted with a grey dotted line.

The performance of each filtering technique was evaluated across different noise levels, comparing their outputs to the ground truth. The 3D Gaussian and HYPR filters exhibited similar trends, with consistent but moderate underestimations (∼20–35%). The 4D Gaussian filter showed the largest underestimation across most noise levels (∼50–75%), particularly at higher noise levels. The MRI-MRF filter also consistently underestimated, with performance deteriorating significantly at high noise levels (−100.16 at 100 noise factor). In contrast, the IHYPR4D filter overestimated the ground truth, especially at lower noise levels (∼50%).The edNLM filter performed the best overall, closely approximating the ground truth across all noise levels, with minimal error compared to other methods. Finally, the dNLM filter showed moderate underestimations, with errors similar to those of the 4D Gaussian and MRI-MRF filters, see Figure 4.

## Discussion

The aim of this paper was to systematically evaluate various filtering techniques in the context of stimulation-induced changes in glucose metabolism using fPET, with a focus on practically relevant parameters such as test-retest variability, tSNR, and spatial task-based activation. The results present a nuanced understanding of the strengths and weaknesses of each filtering method, providing valuable information to choose the most suitable technique.

## Spatial filtering techniques

The comparison of 3D Gaussian, HYPR, and MRI-MRF L = 10 filters revealed similar performance across all metrics. These similarities, coupled with the additional complexity and processing time of the MRI-MRF filter, emphasize the importance of considering computational efficiency. While the MRI-MRF filter technique shows decent task-induced activation at the group level, it faces challenges due to the absence of a temporal component.^16^ The crucial parameter L (i.e., MRF neighborhood) in MRI-MRF was found to significantly influence its performance, introducing a trade-off between spatial resolution and overall efficacy. When choosing the L parameter, the decision should be guided by the specific requirements of the study, as it has a profound impact on the spatial resolution. While the dNLM showed high spatial task-based activation statistics, its test-retest variability remained average when compared to other spatial filtering techniques. Upon examining the spatial task-based activation (Figures 1 and 2), it appears that the elevated t-statistic values (Table 1) may result from the overestimation of task-based activation (see below, section: Temporal and spatial differences). This further shows that increasing effect sizes do not always signify favorable outcomes when employing certain filtering techniques, as they may also indicate overfitting or other undesirable effects.

**Table 1.** Summarized overview of all examined parameters including Intraclass Correlation Coefficients (ICC), temporal signal to noise ratio (tSNR), peak and mean T-values for the best performing hyperparamters per filter technique.

Filter	ICC	tSNR	Mean T-value	Peak T-value	Sample size calculation
3D Gaussian 6 mm	0.55	**12.28**	3.61	9.33	13 [100%]
3D Gaussian 8 mm	**0.57**	12.09	4.51	10.75	13 [100%]
3D Gaussian 10 mm	0.53	11.91	**5.41**	**11.54**	12 [92.3%]
4D Gaussian 5 Frames	**0.47**	**11.61**	**3.22**	**4.73**	19 [146.1%]
MRI-MRF L = 6 mm	0.09	8.50	**6.34**	11.29	33 [253.8%]
MRI-MRF L = 10 mm	**0.53**	**12.00**	4.80	11.86	15 [115.4%]
MRI-MRF L = 14 mm	0.47	10.61	6.12	**13.12**	17 [130.8%]
HYPR 6 mm	**0.54**	**12.36**	2.46	8.38	17 [130.8%]
HYPR 8 mm	**0.54**	12.05	4.44	10.45	16 [123.1%]
HYPR 10 mm	0.53	11.91	**5.38**	**11.93**	12 [92.3%]
IHYPR4D 7 Frames	**0.56**	**14.27**	**6.13**	**13.51**	16 [123.1%]
edNLM 3 × 5 Frames	**0.61**	12.72	6.22	14.18	**11 [84.6%]**
edNLM 5 × 7 Frames	0.55	**13.74**	**6.66**	**14.23**	12 [92.3%]
dNLM	**0.50**	**13.51**	8.42	**15.43**	15 [115.4%]

Furthermore, the estimated sample size per filter is also listed as absolute numbers and relative to the 3D Gaussian filter in %. The values represent an average of both difficulty levels and all three regions of interest encompassing the frontal eye field, intraparietal sulcus and the occipital cortex. Bold values indicate the best performing hyperparamters within each filter technique. Separate values for each brain region and task difficulty are available in supplemental tables 1–4.

## Spatiotemporal filtering techniques

While the 4D Gaussian, IHYPR4D and edNLM filter techniques employ a similar sliding-window approach with comparable spatial and temporal parameters, pronounced differences in the outcome parameters were found. The 4D Gaussian filter exhibited suboptimal performance in all categories, as evidenced by its decreased uptake during task and rest, which could be attributed to excessive temporal smoothing over the task effect. This highlights the importance of preserving temporal features. In contrast, the edNLM filter, which emerged as a more versatile choice, employs a local search window and patch-based strategy.^18^ This allows for the preservation of task-induced features while adapting to variations in temporal signals. Application of such an adaptive smoothing kernel, as seen in NLM, is further supported by its ability to maintain sharp edges, by not sampling voxels outside the brain or grey matter. The IHYPR4D filter outperformed the 4D Gaussian filter, which could be attributed to its iterative processing and inclusion of a composite image. The composite image acts similarly to NLM by preserving spatiotemporal features during denoising, while iterative updates help reduce contrast mismatches between the composite and input data.

## Implications of filter selection

While every study poses a unique hypothesis, it is important to note that each filter has its strengths and weaknesses. The output parameters highlight their relevance in different facets of fPET analyses. The emphasis on tSNR, which is important for single subject analysis and comparing task conditions, highlights the superior performance of the IHYPR4D, MRI-MRF L = 10, dNLM, and edNLM filters. On the other hand, power calculations, which are usually performed using task-based activations (i.e., peak or mean T-values), favored dNLM and edNLM filters, while MRI-MRF also demonstrated decent improvements compared to standard 3D Gaussian filtering. The assessment of ICC for comparing the reliability between multiple measurements emphasized the strengths of HYPR and edNLM filters. Albeit with the caveat of the HYPR filter not taking the temporal component into account, which can be crucial when working with high-temporal resolution fPET data.^7^ Furthermore, the observed variations in sample size requirements across different filtering techniques underscore the impact of methodological choices on statistical power. The 4D Gaussian demonstrated notably lower power, necessitating larger sample sizes compared to the 3D Gaussian filter, which implies increased resource allocation. On the other hand, the HYPR, IHYPR4D, MRI-MRF as well as dNLM exhibited comparable sample size requirements. Notably, the edNLM stood out with the lowest sample size demand, potentially decreasing the sample size needed for a study. Consequently, this reduction may play a vital role when disentangling more nuanced task effects between different conditions. The selection of an appropriate filter and its hyperparameters, therefore, becomes contingent on the specific metric of interest, underlining the need for a nuanced approach based on the study's objectives.

## Temporal and spatial differences

The temporal overview of various filtering techniques offers critical insights into their effects on the fPET signal dynamics. The observed tendency of the MRI-MRF L = 14 and Gaussian 4D filters to excessively smooth the signal in the temporal domain highlights potential drawbacks in capturing both acute changes and the general task effect. Conversely, the Gaussian 3D, HYPR, and MRI-MRF L = 10 filters demonstrated a more balanced approach, reducing peaks while maintaining signal stability. The dNLM filter demonstrated a considerably smoother global time course, suggesting a potential noise reduction but at the cost of reduced differentiation between rest and task periods. However, this excessive temporal smoothing, driven by the large temporal window, appears to be influenced more by baseline uptake, which is considerably higher, rather than the task-induced uptake changes. Additionally, these factors contribute to an overestimation of task activation throughout the brain and have been demonstrated to dampen the magnitude of task-induced effects. Consequently, this could result in a lack of spatial specificity, despite producing high task-specific group statistic metrics. The edNLM filter further underscored the ability to maintain the amplitude of task-induced changes, while providing a time course with less noise. Moreover, the MRI-MRF L = 14s underestimation of both global and task-active tracer uptake indicates that using a large neighborhood parameter is not optimal for fPET. The MRI-MRF L = 10 shows a more similar tracer uptake when compared to other filtering techniques. Furthermore, both NLM techniques and MRI-MRF L = 14 show markedly improved T-values at peak level and across the entire task-active region in comparison to other filter techniques. All in all, filtering techniques that overly smooth the time course are not suitable in high temporal resolution fPET frameworks.^7^

## Processing time

A consideration of processing time provided valuable insights into the feasibility of implementing different filters on typical hardware configurations. While 3D Gaussian smoothing proved to be a quick and efficient option, the high processing times associated with NLM and MRI-MRF filters or the requirement for dedicated processing servers underlined the need for careful consideration in resource-constrained environments. The HYPR, IHYPR4D, and 4D Gaussian smoothing fell within a moderate processing time range, making them more practical for a broader range of applications and hardware options. Although all filters were programmed in MATLAB, optimizing the code or utilizing other programming languages capable of more efficient data processing could significantly decrease the runtime of complex filtering techniques, potentially rendering them more suitable for general use.

## Post-processing denoising

In this work, we focus exclusively on post-processing denoising methods, recognizing that while reconstruction-based approaches are vast and have shown promise in denoising, they often require modifications to the image acquisition process or reconstruction algorithms, which may not be feasible in all scenarios. Post-processing denoising, in contrast, can be applied retrospectively to existing data without altering acquisition parameters, making it highly flexible and adaptable across different studies and imaging platforms. Furthermore, post-processing techniques allow for targeted noise reduction and artifact removal, while preserving the original temporal and spatial structure of the data. This flexibility is particularly important in fPET imaging, where subtle signal changes related to external stimulations require precise improvements to SNR without compromising data integrity.

## Simulations

The results from the simulation and real-life data showed both consistencies and contrasts in filter performance. In both analyses, the edNLM filter consistently demonstrated superior performance. In simulations, edNLM closely approximated the ground truth across noise levels, while in real-life data, it achieved the highest ICC, tSNR, and spatial activation metrics. Similarly, the HYPR filter exhibited competitive performance in both scenarios, though it underperformed compared to edNLM in real data. The 4D Gaussian filter consistently underperformed in both datasets, showing the largest underestimations in simulations and the lowest ICC and tSNR values in real data. Interestingly, the IHYPR4D filter showed moderate performance in both settings, with overestimations in simulations and ICC values comparable to edNLM for its best variant. MRI-MRF filters showed mixed results; the best configuration showed competitive spatial activation and ICC values in real data but displayed underestimations in simulations. Overall, while the trends were aligned in highlighting edNLM’s robustness and 4D Gaussian’s limitations, real-life data also revealed nuances in tSNR and task-based activation that were less evident in simulations, reflecting additional challenges in real-world signal variability.

## Limitations

While the absence of a ground truth method can be seen as a limitation, we relied on the 3D Gaussian filter as a reference, due to its widespread usage in multiple modalities. Furthermore, the definition of task-active ROIs using a conjunction of signals may introduce a potential source of bias, but on the other hand makes it less dependent on fPET alone. The limited sample size, although typical for PET studies, poses constraints, for the future assessment of advanced techniques such as deep learning,^30,31^ where larger datasets are often required. The use of standard algorithm parameters from previous literature, while practical, may not capture the full spectrum of parameter space, limiting the generalizability of the findings. However, an exhaustive evaluation is limited by the computational expense of several filters. The structural component was not included in filters other than MRI-MRF, representing another limitation. This could potentially influence the overall performance of the filters, particularly in scenarios where structural information is crucial. Finally, we note that applying a low-pass filter, which is part of the standard fPET preprocessing pipeline,^1,2,4,5,7,32^ after denoising may reduce the distinct temporal characteristics introduced by each denoising method. Still, the same filter was applied throughout, thus, imposing a similar effect for each denoising method.

## Conclusion

We aimed to provide an overview of the strengths and limitations of various filtering approaches in the context of fPET studies. The choice of filtering technique should be tailored to the specific parameter of interest in improving the hypothesis as each method has specific trade-offs. The 3D Gaussian filter reduces high-frequency noise but may also blur small spatial features, while HYPR can bias contrast at time points that differ greatly from the composite image. Determining the optimal iterations for IHYPR4D can be challenging, impacting consistency, and NLM methods are computationally intensive, potentially limiting their use with large datasets. The edNLM filter emerges as a promising compromise, exhibiting the best overall performance across various metrics. However, it may be less suitable for specific use cases such as when minimal processing power is available or only a specific parameter is of interest. Following closely are the MRI-MRF L = 10 and the HYPR filters, each offering unique advantages. The 3D Gaussian filter stands out for its efficient processing time and respectable performance, still making it a viable option in scenarios where computational efficiency is paramount.

## Supplemental Material

sj-pdf-1-jcb-10.1177_0271678X251325668 - Supplemental material for Optimal filtering strategies for task-specific functional PET imaging Supplemental material, sj-pdf-1-jcb-10.1177_0271678X251325668 for Optimal filtering strategies for task-specific functional PET imaging by Murray Bruce Reed, Magdalena Ponce de León, Sebastian Klug, Christian Milz, Leo Robert Silberbauer, Pia Falb, Godber Mathis Godbersen, Sharna Jamadar, Zhaolin Chen, Lukas Nics, Marcus Hacker, Rupert Lanzenberger and Andreas Hahn in Journal of Cerebral Blood Flow & Metabolism

## Data Availability

Raw data will not be publicly available due to reasons of data protection. Processed data and custom code can be obtained from the corresponding author with a data-sharing agreement, approved by the departments of legal affairs and data clearing of the Medical University of Vienna.
