# Supplementary material for: Optimal filtering strategies for task-specific functional PET imaging
Source: J Cereb Blood Flow Metab. 2025 Apr 2;45(9):1760–73. doi: 10.1177/0271678X251325668 (PMC12409040; doi:10.1177/0271678X251325668)
Supplement: sj-pdf-1-jcb-10.1177_0271678X251325668 - Supplemental material for Optimal filtering strategies for task-specific functional PET imaging [file sj-pdf-1-jcb-10.1177_0271678X251325668.pdf]

Optimal filtering strategies for task-specific functional PET imaging

Murray Bruce Reed[1,2]. Magdalena Ponce de León[1,2]. Sebastian Klug[1,2]. Christian Milz[1,2]. Leo Silberbauer[1,2]. Pia Falb[1,2]. Godber Mathis Godbersen[1,2]. Sharna Jamadar[3,4,5]. Zhaolin Chen[3] Lukas Nics[6]. Marcus Hacker[6]. Rupert Lanzenberger[1,2]. Andreas Hahn[1,2 #]

[1]Department of Psychiatry and Psychotherapy. Medical University of Vienna. Austria

[2]Comprehensive Center for Clinical Neurosciences and Mental Health (C3NMH). Medical University of Vienna. Austria

[3]Monash Biomedical Imaging. Monash University. Melbourne. Victoria. Australia

[4]Australian Research Council Centre of Excellence for Integrative Brain Function. Melbourne. Victoria. Australia

[5]School of Psychological Sciences, Monash University. Melbourne. Victoria. Australia.

[6]Department of Biomedical Imaging and Image-guided Therapy. Division of Nuclear Medicine. Medical University of Vienna. Austria

Supplementary Material

For submission to:

Journal of Cerebral Blood Flow & Metabolism

Running Title: Optimal filters for fPET

# Correspondence to:
Assoc. Prof. PD. Dr. Andreas Hahn. MSc
Email: andreas.hahn@meduniwien.ac.at
ORCID: https://orcid.org/0000-0001-9727-7580
Medical University of Vienna. Department of Psychiatry and Psychotherapy. Austria

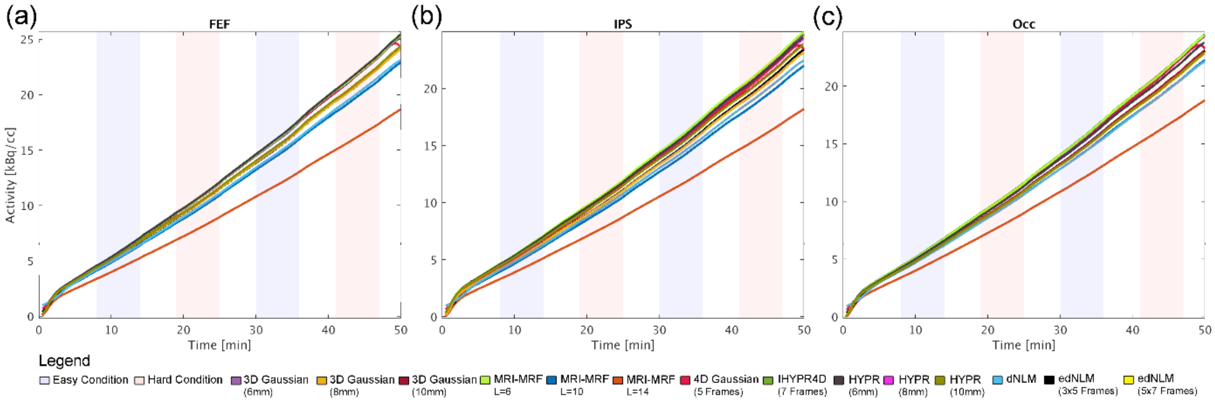

Supplementary Figure 1: Overview of time activity curves (averaged across participants) obtained with for the best performing hyperparamters per filter technique for a subset of participants whose task difficulty was ordered as easy-hard-easy-hard (n = 10). extracted from the (a) frontal eye field (FEF). (b) intraparietal sulcus (IPS) and (c) occipital cortex (OCC).

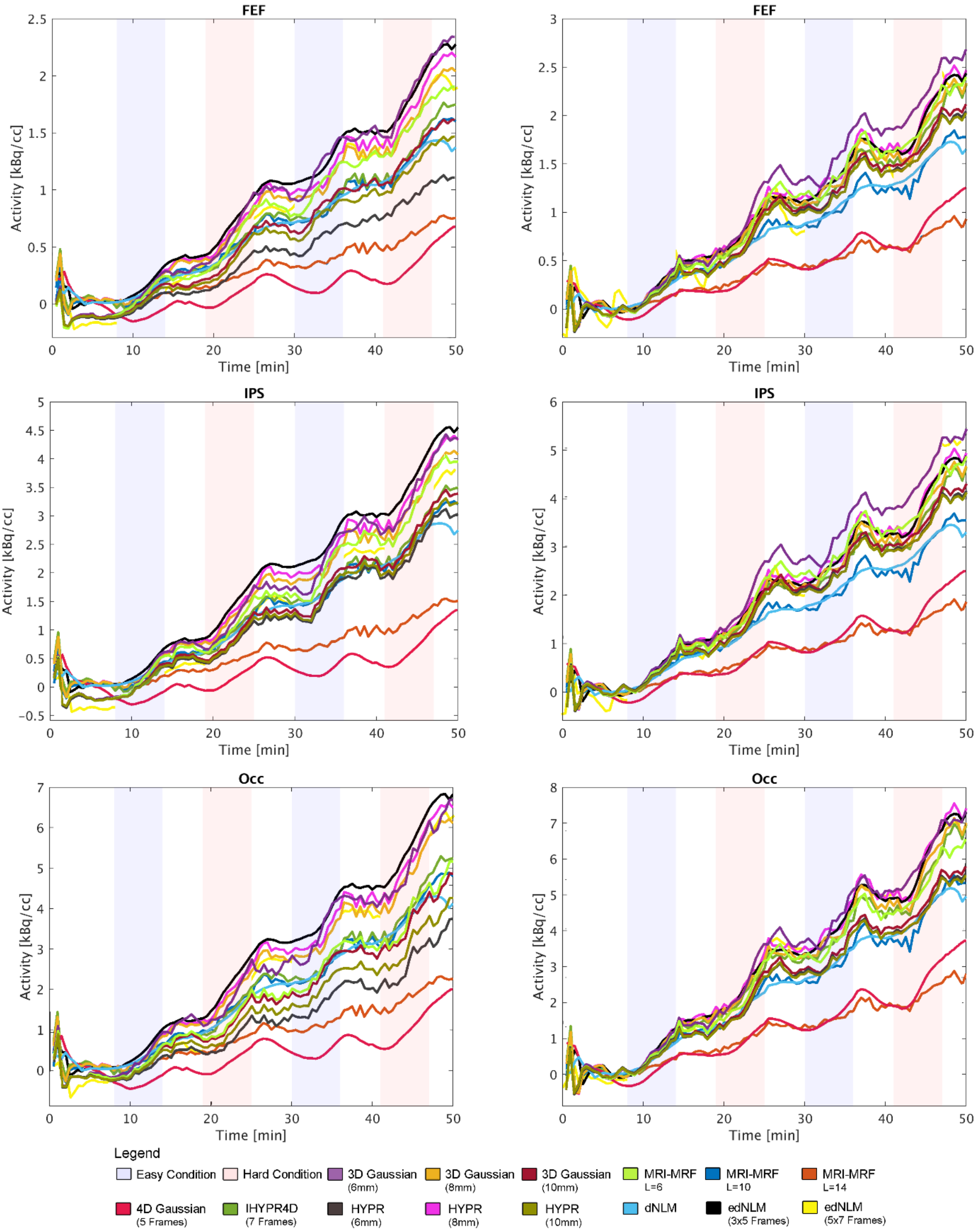

Supplementary Figure 2: Overview of task-specific regressors (Hard > BL) obtained for the best performing hyperparamters per filter technique for two representative participants (left and right columns. respectively). Region of interest included the frontal eye field (FEF). intraparietal sulcus (IPS) and the occipital cortex (OCC).

Filter	FEF		IPS		OCC		Mean
	Easy	Hard	Easy	Hard	Easy	Hard	
**3D Gaussian 6mm**	0.51	0.52	0.24	0.73	0.77	0.53	0.55
**3D Gaussian 8mm**	0.50	0.65	0.53	0.76	0.34	0.65	0.57
**3D Gaussian 10mm**	0.39	0.51	0.21	0.72	0.78	0.55	0.53
**4D Gaussian 3 Frames**	0.43	0.39	0.39	0.11	0.21	0.38	0.32
**4D Gaussian 5 Frames**	0.34	0.40	0.15	0.66	0.72	0.53	0.47
**4D Gaussian 7 Frames**	0.38	0.31	0.34	0.01	0.24	0.29	0.26
**MRI-MRF L=6mm**	0.07	0.05	0.08	0.13	0.11	0.12	0.09
**MRI-MRF L=10mm**	0.40	0.52	0.22	0.72	0.79	0.55	0.53
**MRI-MRF L=14mm**	0.40	0.51	0.04	0.71	0.73	0.45	0.47
**HYPR 6mm**	0.46	0.52	0.24	0.73	0.77	0.53	0.54
**HYPR 8mm**	0.44	0.52	0.23	0.73	0.78	0.54	0.54
**HYPR 10mm**	0.39	0.51	0.21	0.72	0.78	0.55	0.53
**IHYPR4D 3 Frames**	0.40	0.50	0.26	0.72	0.73	0.51	0.52
**IHYPR4D 5 Frames**	0.37	0.47	0.27	0.72	0.72	0.49	0.51
**IHYPR4D 7 Frames**	0.39	0.53	0.37	0.70	0.76	0.61	0.56
**edNLM 3x3 Frames**	0.48	0.51	0.32	0.70	0.76	0.57	0.56
**edNLM 3x5 Frames**	0.47	0.54	0.68	0.80	0.47	0.70	0.61
**edNLM 3x7 Frames**	0.30	0.64	0.38	0.56	0.74	0.60	0.54
**edNLM 5x3 Frames**	0.45	0.51	0.32	0.69	0.76	0.59	0.55
**edNLM 5x5 Frames**	0.47	0.56	0.18	0.71	0.76	0.38	0.51
**edNLM 5x7 Frames**	0.42	0.50	0.34	0.72	0.73	0.59	0.55
**dNLM**	0.43	0.56	0.34	0.80	0.27	0.63	0.50

Supplementary Table 1: Detailed overview of Intraclass Correlation Coefficients separately for each region of interest and both task difficulties. The total average value is the same as in table 1 of the main text. These regions comprise the frontal eye field (FEF). intraparietal sulcus (IPS) and the occipital cortex (OCC).

Filter	FEF		IPS		OCC		Mean
	Easy	Hard	Easy	Hard	Easy	Hard	
**3D Gaussian 6mm**	10.37	11.31	9.12	15.91	13.44	13.52	12.28
**3D Gaussian 8mm**	10.26	11.31	8.96	15.57	13.22	13.23	12.09
**3D Gaussian 10mm**	10.10	11.31	8.81	15.22	13.02	12.99	11.91
**4D Gaussian 3 Frames**	9.35	9.17	10.27	10.78	11.54	11.52	10.44
**4D Gaussian 5 Frames**	9.80	11.57	8.78	12.21	13.70	13.59	11.61
**4D Gaussian 7 Frames**	9.39	9.15	10.54	10.96	11.88	9.55	10.25
**MRI-MRF L=6mm**	8.61	8.56	7.78	8.97	8.66	8.41	8.50
**MRI-MRF L=10mm**	9.73	11.41	9.00	15.41	13.53	12.91	12.00
**MRI-MRF L=14mm**	7.70	10.13	8.45	12.50	12.84	12.06	10.61
**HYPR 6mm**	10.51	11.24	9.39	15.38	13.72	13.94	12.36
**HYPR 8mm**	10.22	11.29	8.91	15.48	13.22	13.15	12.05
**HYPR 10mm**	10.10	11.31	8.81	15.22	13.02	12.99	11.91
**IHYPR4D 3 Frames**	9.85	11.45	9.06	14.68	12.58	12.62	11.71
**IHYPR4D 5 Frames**	10.03	11.61	9.08	14.57	12.52	12.50	11.72
**IHYPR4D 7 Frames**	13.78	14.41	11.61	16.68	14.64	14.50	14.27
**edNLM 3x3 Frames**	10.57	11.68	9.40	15.76	13.26	13.28	12.32
**edNLM 3x5 Frames**	11.21	11.99	10.06	16.23	13.19	13.62	12.72
**edNLM 3x7 Frames**	11.11	10.57	9.72	17.34	12.08	13.18	12.33
**edNLM 5x3 Frames**	10.58	11.91	9.40	15.61	13.21	13.11	12.30
**edNLM 5x5 Frames**	9.58	11.65	6.45	14.35	12.89	10.44	10.89
**edNLM 5x7 Frames**	13.47	13.53	10.79	16.78	13.93	13.92	13.74
**dNLM**	13.39	13.78	10.74	14.63	14.62	13.91	13.51

Supplementary Table 2: Detailed overview of temporal signal to noise ratio (tSNR) estimated separately for each region of interest and task difficulty. The total average value is the same as in table 1 of the main text. The three regions include the frontal eye field (FEF). intraparietal sulcus (IPS) and the occipital cortex (OCC).

Filter	FEF		IPS		OCC		Mean
	Easy	Hard	Easy	Hard	Easy	Hard	
**3D Gaussian 6mm**	3.67	3.38	2.77	4.36	3.77	3.71	3.61
**3D Gaussian 8mm**	4.60	4.18	3.43	5.53	4.70	4.60	4.51
**3D Gaussian 10mm**	5.29	5.07	4.13	6.78	5.58	5.60	5.41
**4D Gaussian 3 Frames**	1.12	1.07	1.48	1.31	1.71	1.69	1.40
**4D Gaussian 5 Frames**	2.59	2.18	2.39	4.65	3.80	3.69	3.22
**4D Gaussian 7 Frames**	1.03	1.04	1.50	1.06	1.43	1.28	1.22
**MRI-MRF L=6mm**	6.37	6.10	5.82	6.92	6.50	6.32	6.34
**MRI-MRF L=10mm**	4.54	4.48	3.92	5.61	5.00	5.26	4.80
**MRI-MRF L=14mm**	5.06	5.58	5.43	6.87	6.32	7.44	6.12
**HYPR 6mm**	2.57	2.29	1.93	2.91	2.54	2.53	2.46
**HYPR 8mm**	4.51	4.14	3.38	5.43	4.64	4.53	4.44
**HYPR 10mm**	5.36	4.96	4.11	6.73	5.58	5.55	5.38
**IHYPR4D 3 Frames**	4.41	4.07	3.25	5.35	4.53	4.40	4.34
**IHYPR4D 5 Frames**	4.48	4.16	3.31	5.44	4.61	4.49	4.42
**IHYPR4D 7 Frames**	6.17	5.72	4.87	7.26	6.42	6.37	6.13
**edNLM 3x3 Frames**	3.96	3.56	2.92	4.58	4.03	3.96	3.83
**edNLM 3x5 Frames**	6.27	5.65	5.07	7.18	6.51	6.63	6.22
**edNLM 3x7 Frames**	4.75	4.39	3.66	5.34	4.79	4.77	4.62
**edNLM 5x3 Frames**	5.06	4.57	3.81	6.11	5.19	5.16	4.98
**edNLM 5x5 Frames**	3.93	4.08	3.02	4.27	4.34	3.90	3.92
**edNLM 5x7 Frames**	6.94	5.90	5.19	8.15	6.76	7.04	6.66
**dNLM**	8.12	7.48	6.86	10.30	8.40	9.34	8.42

Supplementary Table 3: Detailed overview of the mean T-value extracted for each region of interest and task difficulty separately. The average value is the same as in table 1 of the main text. The three regions include the frontal eye field (FEF). intraparietal sulcus (IPS) and the occipital cortex (OCC).

Filter	FEF		IPS		OCC		Mean
	Easy	Hard	Easy	Hard	Easy	Hard	
**3D Gaussian 6mm**	10.51	9.21	7.23	10.09	9.69	9.23	9.33
**3D Gaussian 8mm**	11.63	9.78	9.04	11.58	11.50	10.99	10.75
**3D Gaussian 10mm**	10.40	12.52	8.95	12.73	11.96	12.70	11.54
**4D Gaussian 3 Frames**	4.07	2.62	3.23	4.23	3.01	2.46	3.27
**4D Gaussian 5 Frames**	3.88	4.86	2.77	5.70	5.02	6.16	4.73
**4D Gaussian 7 Frames**	4.55	2.35	3.14	4.59	3.27	3.34	3.54
**MRI-MRF L=6mm**	13.22	10.32	10.16	11.65	11.17	11.22	11.29
**MRI-MRF L=10mm**	12.30	11.05	8.93	13.65	12.25	12.97	11.86
**MRI-MRF L=14mm**	9.97	11.96	10.75	15.26	12.54	18.23	13.12
**HYPR 6mm**	10.10	7.27	6.42	10.87	7.76	7.84	8.38
**HYPR 8mm**	10.76	9.43	8.65	11.66	11.03	11.15	10.45
**HYPR 10mm**	11.02	11.01	9.93	13.48	12.70	13.42	11.93
**IHYPR4D 3 Frames**	12.02	9.76	8.80	11.29	11.64	10.43	10.66
**IHYPR4D 5 Frames**	12.80	9.46	8.87	11.12	11.82	10.62	10.78
**IHYPR4D 7 Frames**	15.41	13.28	10.63	12.92	14.21	14.64	13.51
**edNLM 3x3 Frames**	11.16	11.01	7.36	9.85	10.49	10.63	10.08
**edNLM 3x5 Frames**	13.13	12.04	10.47	13.97	17.26	18.19	14.18
**edNLM 3x7 Frames**	12.57	10.44	8.30	11.75	13.88	13.48	11.74
**edNLM 5x3 Frames**	14.15	10.40	8.14	12.17	12.30	13.33	11.75
**edNLM 5x5 Frames**	13.61	10.06	10.01	12.62	14.23	13.06	12.26
**edNLM 5x7 Frames**	16.44	11.09	9.65	16.92	13.48	17.79	14.23
**dNLM**	12.84	13.69	11.97	16.59	16.31	21.18	15.43

Supplementary Table 4: Detailed overview of the peak T-value extracted for each region of interest and task difficulty separately. The average value is the same as in table 1 of the main text. The three regions encompass the frontal eye field (FEF). intraparietal sulcus (IPS) and the occipital cortex (OCC).
